# A case of tuberculosis in the cavitary lesion suggestive of neoplasm

**DOI:** 10.1590/0037-8682-0229-2025

**Published:** 2025-09-29

**Authors:** Cemile Altınkaya, Adem Karaman

**Affiliations:** 1 Department of Radiology, Ataturk University School of Medicine, Erzurum, Turkey.

A 60-year-old man was admitted to the chest disease outpatient clinic with shortness of breath, hemoptysis, and cough. Chest radiography revealed a radiopaque, round, and cavitary lesion in the lower lobe of the right lung (Figure 1). The patient underwent non-contrast thorax imaging and positron emission tomography/computed tomography (PET-CT) imaging. PET-CT showed a 61×52 mm hypermetabolic cavitary mass lesion in the lower lobe of the right lung, with a wall thickness of 13 mm at the thickest part and internal surface irregularities (Figure 2 and 3). A thoracic Tru-Cut biopsy was performed because malignancy was suspected.

**FİGURE 1:**
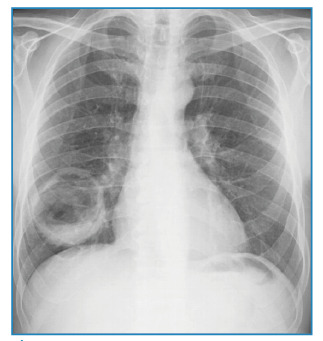
Radiopaque, round-shaped cavitary lesion in the lower lobe of the right lung on chest radiography.

**FİGURE 2:**
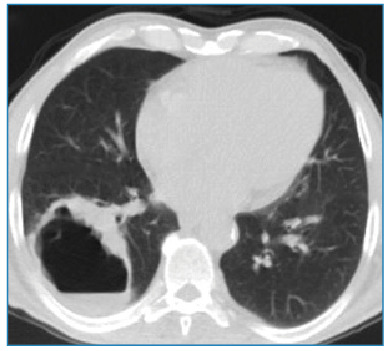
Cavitary lesion in the lower lobe of the right lung on non-contrast thorax computed tomography images.

**FİGURE 3:**
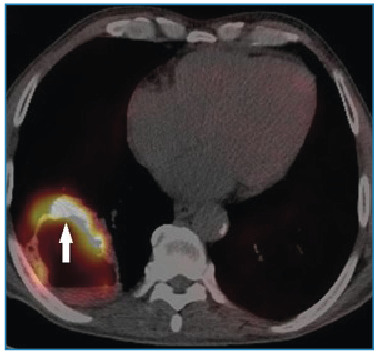
Positron emission tomography/computed tomography imaging shows hypermetabolic involvement.

The pathological results were nonspecific. Because the sputum culture from the patient was positive for M. tuberculosis, this lesion was evaluated as a cavitary lesion due to tuberculosis.

CT plays a crucial role in differentiating between benign and malignant cavitary pulmonary lesions. Radiological features suggestive of malignancy include cavity wall thickness >5 mm, irregular or nodular inner margins, presence of air-fluid levels, and increased metabolic activity on PET-CT^1^ ^-^ ^3^.

In cavitary mass lesions with malignant criteria, tuberculosis-related cavitary lesions may also exhibit hypermetabolic uptake on PET-CT^4^.
